# Water-flow stress differentially affects the morphological, anatomical, and mechanical traits of *Osmunda* x *intermedia* (Osmundaceae) populations growing inside and outside the river curve

**DOI:** 10.3389/fpls.2025.1651616

**Published:** 2025-10-06

**Authors:** Shunsuke Hara, Masayuki Shiba, Tatsuya Fukuda

**Affiliations:** Graduate School of Integrative Science and Engineering, Tokyo City University, Tokyo, Japan

**Keywords:** acclimation, biomechanics, lamina–petiole relationship, pinnule, rheophyte

## Abstract

The curve of a river bed creates a difference in the speed of water flow inside and outside this curve, indicating that plants growing along the river experience differential water-flow stresses during sudden floods caused by heavy rains. In this study, we conducted morphological, anatomical, and mechanical analyses using *Osmunda* x *intermedia* (Honda) Sugim. (Osmundaceae), a hybrid of *Osmunda japonica* Thunb. and the rheophytic *O. lancea* Thunb., growing inside and outside the river curve to elucidate the plant traits influenced by differential water-flow stresses. The external morphological analysis revealed that the *O.* x *intermedia* populations growing both inside and outside the river curve exhibited values intermediate between those of the parent species. However, the results of the anatomical and mechanical analyses of the petioles of the hybrid species did not necessarily reveal values intermediate between those of the parent species; however, in the hybrid species, the cell wall volume per unit volume was related to petiole strength, and the cell wall volume per unit volume of the hybrid population growing inside the river curve was significantly higher than that in the parent species or the hybrid population outside the river curve. In addition, the flexibility of petioles in the hybrid population growing outside the curve was associated with a lower cell wall density in the sterome than in that inside the curve, which may cause elastic bending that bends the cells further because of thinner cell walls. The results obtained in our study revealed that *O.* x *intermedia* adapts to different water-flow stresses through complex anatomical and mechanical changes that cannot be determined from external morphology alone.

## 1 Introduction

Environmental adaptation is an important source of plant biodiversity contributing to the evolution of phenotypic diversity in response to ecological changes (Grant, 1981; Givnish, 2015), and this phenotypic diversity is recognized as an adaptation to various environmental factors and stresses (Rowe and Speck, 2005; Santiago and Wright, 2007; Gardiner et al., 2016; Anest et al., 2021). Many studies have reported that physiological stress, in particular, is one of the factors that significantly alters plant morphology in various environments (Hayakawa et al., 2012; Tunala et al., 2012; Ohga et al., 2012b; Kumekawa et al., 2013; Sunami et al., 2013; Ishii et al., 2022; Shiba et al., 2022a, 2022b, 2022c; Takizawa et al., 2022, 2023; Marui et al., 2023; Endo et al., 2025).

Mechanical loads on plants considerably impact their growth, morphology, and ecology. For example, many studies have discussed the interactions of wind stress with plants and the effects of wind loads on plants (de Langre, 2008; Mitchell, 2013). Niklas (1996) reported that sugar maple leaves sampled from young trees in wind-exposed areas have smaller leaf blades and more flexible petioles than in leaves sampled from protected areas, indicating that wind affects both leaves and petioles in sugar maples. Based on wind-induced bending and twisting stress analyses in red oak, American sycamore, yellow poplar, and sugar maple, Louf et al. (2018) reported that the ability of leaves to reduce wind stress at the stem–petiole junction can be achieved by locating the twisting area closer to the lamina. They further described that the strain decreases closer to the stem even if the stress is constant throughout the petiole. Moreover, using certain coastal tree species, Meguro and Miyawaki (1994) reported that the strain energy per unit volume of branches was higher in individuals grown under strong winds than in those grown under weak winds. In addition to such studies on trees, studies have also been conducted on herbaceous plants; Shiba et al. (2023) revealed that the adaptation process of *Farfugium japonicum* (L.) Kitamura (Asteraceae) to a strong wind environment reduces lamina size and shortens petioles. Notably, this species has been shown to shorten its scape under similar wind stress conditions, although the scape has a mechanically stronger structure than that of the petiole (Shiba et al., 2024b). These studies provide detailed reports on the adaptive morphology of plants in response to wind stress.

Irregular flooding can be an important stressor for plants that do not inherently have or cannot develop the characteristics to survive under submerged conditions. Flooding after heavy rainfall caused by hurricanes, cyclones, and typhoons (Blom and Voesenek, 1996) considerably impacts plant survival (Vervuren et al., 2003; van Eck et al., 2004). Plants along rivers are exposed to flash floods, a major source of mechanical stress, as a strong selective pressure. Therefore, they have lanceolate or cuneate laminae to reduce the stress caused by sudden flooding and strong river currents after heavy rain (van Steenis, 1981). Such morphological characteristics of the laminae have been reported in various taxa ranging from ferns to angiosperms (Yamada et al., 2011; Ohga et al., 2012a; Ueda et al., 2012; Yokoyama et al., 2012; Kumekawa et al., 2013; Matsui et al., 2013; Shiba et al., 2021). Among them is *Osmunda lancea* Thunb. (Osmundaceae), which grows on riverbanks subject to flooding after heavy rains; therefore, this species is a rheophyte with narrow-lanceolate pinnules resistant to relatively fast river currents. A comparative study of *O. lancea* and its closely related inland species, *O. japonica* Thunb., indicated a strong correlation between the gross morphology and anatomy of pinnules (Imaichi and Kato, 1992, 1993). Furthermore, Shiba and Fukuda (2024) reported that the petioles of *O. lancea* have the flexibility to reduce stresses caused by swift river currents, and similar results were reported for the stipes of *O. lancea* (Shiba and Fukuda, 2025). In particular, regarding the relationship between *O. lancea* and *O. japonica*, the morphology of *O.* x *intermedia* in environments with less water-flow stress than riverine habitats where *O. lancea* typically grows remains to be elucidated. Some morphological and molecular studies have reported *Osmunda* x *intermedia* (Honda) Sugim, a hybrid between *O. lancea* and *O. japonica* with morphological intermediacy in pinnule shape (Kato, 2007; Yatabe et al., 1999), with many of its habitats overlapping with those of *O. lancea* (Kato, 2007). Hybrids often exhibit intermediate values for both morphological and mechanical properties (Shiba et al., 2024a), and backcrossing through hybrids has been reported to exceed the values of both parent species (Rieseberg et al., 2000). Tsutsumi et al. (2013) reported that *O.* x *intermedia* did not grow in the riverside zone but on the top of the mountain on Kozushima Island in the Izu Islands, Japan, suggesting that similar pinnule forms found along rivers may result from distinct environmental factors such as wind or flooding. This suggests that the adaptation of *O.* x *intermedia* to a wide range of mechanical stresses can lead to the detection of mechanical stresses of varying intensities by analyzing this species. However, whether sites with differing mechanical stress exist within otherwise similar environments remains unclear.

River meanders are among the most common river morphology patterns. A river meander is one of a series of regular sinuous curves in a river or waterway channel, formed when the waterway erodes sediment along its outer concave banks or cliffs and deposits it along the inner convex banks by water velocity and the associated physical forces (Allan and Castillo, 2007). The meandering course results from the combined processes of erosion and deposition (Chitale, 1970). The significance of regular loops in rivers has attracted the attention of researchers from the fields of hydrodynamics, morphodynamics (Seminara, 1998, 2006), and petroleum engineering (Swanson, 1993). In addition, the geomorphological studies of meandering rivers have elucidated the relationship between the planar characteristics of meanders and the riverbed morphology based on the main features of meanders through field surveys (Leopold and Wolman, 1960; Allen, 1965; Chitale, 1970; Nanson and Hickin, 1983; Carson and Lapointe, 1983; Thorne and Furbish, 1995) and laboratory experiments (Zimmerman and Kennedy, 1978; Kinoshita and Miwa, 1974; Whiting and Dietrich, 1993a, 1993b). Furthermore, riparian zones are among the most productive and valuable natural resources worldwide because they support numerous ecological services, such as plant species diversity and wildlife habitat (Sakio, 1997). For example, riparian ecology studies have revealed that large-scale natural forest disturbances and primary succession in the lowland tropical rainforests of the Peruvian Amazon are caused by lateral erosion and channel changes in meandering rivers. Moreover, primary succession on the newly deposited fluvial soils of meandering rivers is the main factor generating and maintaining the high between-habitat species diversity characterizing the area (Salo et al., 1986). In addition, a few studies have reported that the formation and development of meandering streams are closely linked to the stabilizing effects of riparian vegetation, such as bank reinforcement by plant roots and vegetation-promoted production and retention of soil silt (Gibling and Davies, 2012; Davies et al., 2020). Therefore, studies have been conducted on river meandering, vegetation, and forests, as well as on soil heterogeneity arising from the dynamic processes of alluvial plains, including variation in water saturation and soil stability (e.g., Erskine et al., 2009; Stella et al., 2011). Focusing on a single bend in a meandering river, the flow velocity of the rivers is higher on the outside of the curve than on the inside, causing plants growing along both sites to experience different water-flow stresses, particularly during floods caused by sudden heavy rains.

**Figure 1:**
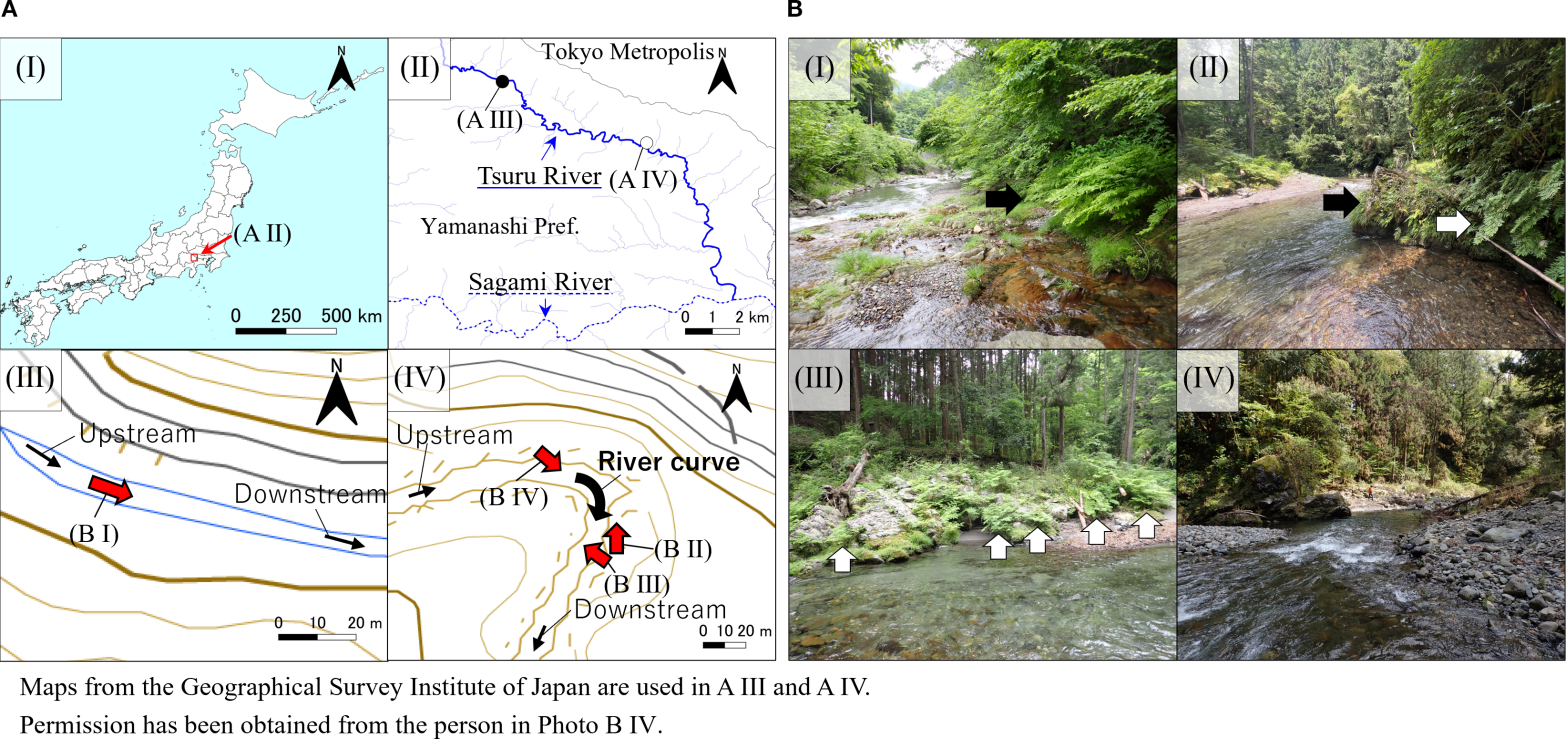
**(A)** Sampling locations used in our study, and **(B)** photographs of the survey area. The red arrows indicate the shooting directions shown in **(B)**. The black arrows indicate *Osmunda japonica* (I) and *O. lancea* (II), while the white arrows indicate the outside (II) and inside (III) of *O*. x *intermedia*. AIII and AIV adapted from the Geospatial Information Authority of Japan (GSI), based on GSI Maps, licensed under the GSI Map Copyright License, https://maps.gsi.go.jp/.

Research on the effect of water-flow stress strength on plant morphology along rivers has been conducted by comparing plant populations in each river. Yamada et al. (2011) reported a relationship between the presence or absence of dams and lamina size by analyzing the leaf morphologies of *Aster microcephalus* (Miq.) Franch. et Sav. var. *ripensis* Makino (Asteraceae), suggesting that water volume regulation through dams reduces the water-flow stress on plants downstream. Sakaguchi et al. (2021) also discussed the variation in lamina size of *Solidago yambaruensis* S. Sakaguchi et Mot. Ito. (Asteraceae) with the differences in water volume between the rivers. However, because these studies compared plant populations between different rivers, they were unable to detect the effects of different vegetation types in each river, as well as the effects of water flow stress, such as flow rate and flow velocity, on plants along the river, suggesting that it was difficult to demonstrate the effects of water flow stress on plant morphology. Cosmo et al. (2024) also reported that plant phenotypic plasticity is highly adaptive to the selective and fluctuating conditions of riparian environments. To solve this problem, therefore, comparing plant communities outside and inside the bends would more accurately reflect the effects of water flow stress on plant morphology and mechanical properties. Our preliminary survey revealed the presence of *O.* x *intermedia* on both sides of a river approximately 5 m wide, with a curvature radius of approximately 30 m (**Figure 1**). This river flowed through a gap in a mixed forest composed of cedar and cypress, and common plant species such as *O.* x *intermedia* and *Phragmites japonicus* Steud. (Poaceae) grew along the river on both banks, and *Rumex japonicus* Houtt. and *Fallopia japonica* (Houtt.) Ronse Decr. (Polygonaceae) and *Hydrangea involucrata* Siebold (Hydrangeaceae) toward the forest margin on each bank. Therefore, we can hypothesize that they exhibit different adaptive modes to different intensities of water flow stress, and the analysis of *O.* x *intermedia* on both banks at this area will reveal adaptive patterns to the strength of water flow stress. Thus, the aim of this study was to elucidate the adaptation pattern of *O.* x *intermedia* in response to differences in water flow stress by comparing its morphological, anatomical, and mechanical characteristics.

## 2 Materials and methods

### 2.1 Plant materials

**Figure 2:**
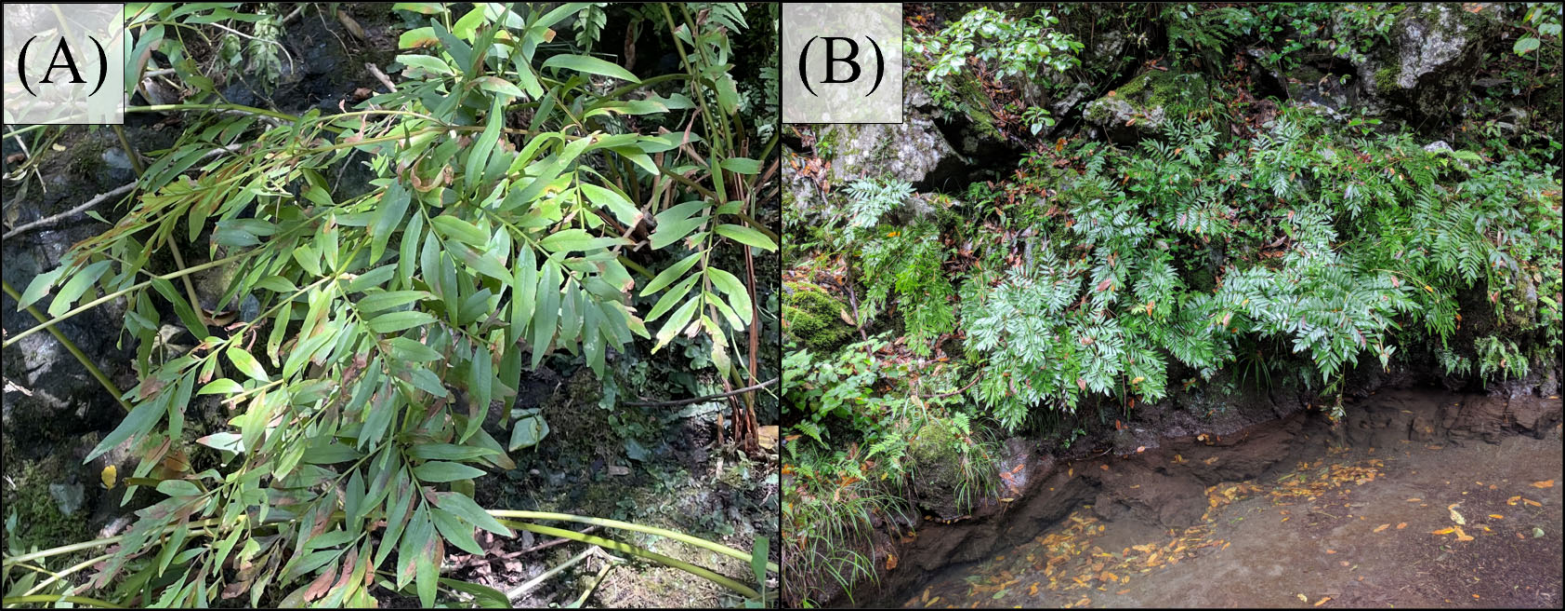
*Osmunda* x *intermedia* populations growing **(A)** inside and **(B)** outside the river curve.

The *O. lancea*, *O. × intermedia*, and *O. japonica* specimens were collected along the Tsuru River in Uenohara City, Yamanashi Prefecture, Japan (**Figure 1**). The Tsuru River basin has an inland climate with extreme temperature fluctuations, with an average annual temperature of 9–14 °C and annual precipitation of approximately 1,600–2,200 mm, concentrated during the rainy season and typhoon season. *O. × intermedia* was sampled from both the inner and outer banks of the river, whereas *O. lancea* was sampled from the outer bank of the same meandering section of the river (**Figures 1**, **2**). According to Iwatsuki (1995), both parent species can be distinguished based on the shape of the pinnule base—cuneate to acute or truncate—and the width of the widest part of pinnules, less than 10 mm in the former and 10–25 mm in the latter. Thus, individuals with broader pinnules and sharper basal angles than in *O. lancea* and *O. japonica*, respectively, were identified as *O. × intermedia*. *O. japonica* specimens were collected upstream from the same river.

The river is approximately 18 m wide at the sampling site, with a curvature radius of approximately 25 m, measured using maps from the Geospatial Information Authority of Japan. The specimens were cut at the petiole base and wrapped in moistened paper to prevent water loss. They were carefully packed underwater to remove air and transported to the laboratory. Mechanical analyses were performed within 24 h of sampling. All procedures were conducted following the regional and national regulations. All analytical procedures were performed using previously described methods (Shiba and Fukuda, 2024).

### 2.2 Morphological analyses

For morphological analysis, we measured the total lamina area of *O.* x *intermedia* specimens and the basal angle, maximum width, length, and area of their pinnules using the ImageJ software (version 1.54). Leaf index (pinnule length-to-width ratio) was also calculated (Tsukaya, 2002). Petiole length was defined as the distance from the petiole base to the lamina base and was measured using a ruler. The cross-sectional shape of the petiole base was evaluated by measuring its major and minor axes using callipers. Although this approach is simplified, the ratio of the major to minor axes is approximately 1.3, suggesting that the petiole base can be approximated as an ellipse. The cross-sectional area *A* of the petiole base was calculated as follows:


(1) $$\begin{array}{c} A\;=\;\frac{ab\pi }{4}, \end{array}$$

where *a* and *b* represent the major and minor axes of the petiole base, respectively.

Relationships between cross-sectional area *A* and petiole length and between petiole length and lamina area were analyzed.

### 2.3 Mechanical analyses

The span length was adjusted based on the sample thickness to maintain a span-to-depth ratio of 15 to accommodate the testing apparatus specifications. Samples were selected from straight regions near the petiole base. The major and minor axes at the midpoint of each sample were measured using callipers to calculate the cross-sectional area, following the method used for the morphological analysis.

Three-point bending tests were performed using a tabletop tensile/compression tester (MCT-1150; A&D, Tokyo Japan) equipped with a bending jig (JM-B1-500N; A&D, Tokyo Japan). The test speed and sampling frequency were set to 10 mm/min and 50 Hz, respectively.

Bending stress σ and strain ϵ were calculated using the following standard equations for elliptical cross-sections under elastic loading:


(2) $$\begin{array}{c} \sigma =\frac{M}{Z}=\frac{PL}{4Z}, \end{array}$$

(3) $$\begin{array}{c} \epsilon =\frac{48\delta \sigma I}{PL^{3}}\times 100 \end{array},$$

where M denotes the bending moment, Z is the section modulus, P represents the applied load, L is the span length, δ represents the displacement, and I is the second moment of the area. Z and I were calculated under the assumption of an elliptical cross-section as follows:


(4) $$\begin{array}{c} Z=\frac{\pi ab^{2}}{32} \end{array},$$

(5) $$\begin{array}{c} I=\frac{\pi ab^{3}}{64}, \end{array}$$

where *a* and *b* are the major and minor axes, respectively.

The bending stress and strain at fracture were defined as bending strength σmax and breaking strain ϵbreak, respectively. The bending modulus of elasticity (E) was calculated from the stress–strain curve between 0.05% and 0.25% strain using the following equation:


(6) $$\begin{array}{c} E=\frac{\sigma_{0.25}\;-\;\sigma_{0.05}}{\epsilon_{0.25}\;-\;\epsilon_{0.05}}\times 100 \end{array}$$

### 2.4 Cell wall volume per unit volume and anatomical analysis of petioles

The fresh volume *V_fresh_* of the petiole was calculated as follows:


(7) $$\begin{array}{c} V_{fresh}\frac{\pi abl}{4} \end{array}$$

where *a* and *b* are the major and minor axes of the elliptical cross-section, respectively, and *l* is the petiole length.

After volume measurement, the samples were dried in an incubator at 75°C for 3 days, and their dry mass *M*_dry_ was measured using a precision balance (ATX224R, SHIMADZU, Tokyo, Japan).

The cell wall mass per unit volume was then calculated using the following equation:


(8) $$\text{Cell wall mass per unit volume}=\frac{M_{dry}}{V_{fresh}}.$$

Petiole segments not used for mechanical testing were excised and fixed in FAA solution (acetic acid: formalin: 99.5% ethanol: distilled water = 5:5:45:45), and transverse sections were prepared. Cross-sectional images were obtained under a light microscope (CX43, OLYMPUS, Tokyo, Japan) equipped with a camera (Moticam X3-12V, SHIMADZU, Tokyo, Japan) and analyzed using ImageJ.

To calculate the sterome-to-petiole cross-sectional area ratio, cross-sections of petioles were imaged under a light microscope (CX43; OLYMPUS, Tokyo, Japan) equipped with a digital microscope camera (Moticam X3-12V; SHIMADZU, Tokyo, Japan). Both the total petiole area and the sterome area were measured using ImageJ, and the ratio was calculated as sterome area divided by total petiole cross-sectional area, expressed as a percentage. The cell wall area fraction within the sterome was determined by measuring the cell wall area contained in a 50 × 50 µm square region (2,500 µm²), and the fraction was calculated as follows:


(9) $$\text{Cell wall area fraction within the sterome}=\frac{cel\;wall\;area\;within\;2,500\mu m^{2}}{2,500\mu m^{2}}.$$

Longitudinal cut were made near the epidermis using a 0.15 mm razor blade for the vertical anatomical observation of cells. The sections were immersed in a dissociation solution (acetic acid: hydrogen peroxide = 1:1) and incubated at 60°C for 3 days. The dissociated samples were observed under the light microscope, and the sclerenchyma cells were identified based on their morphology (Kijima, 1987). We imaged 30 sclerenchyma cells for each individual, and their lengths were measured using ImageJ. The average cell length was used as the representative value for each individual.

### 2.5 Statistical analyses

All statistical analyses were performed using the software R4.4.2. The normality and variance homogeneity of each measured variable were assessed using the Shapiro–Wilk and Levene’s tests, respectively (p< 0.05, the same applies to the following tests). Group differences were evaluated using one-way analysis of variance (ANOVA) or Welch’s ANOVA, as appropriate, and all *post hoc* comparisons were performed using Tukey’s HSD test. For bivariate comparisons, Pearson’s correlation analysis and an analysis of covariance (ANCOVA) were performed. Based on the ANCOVA results, group differences in slope (estimated using the emtrends function) and adjusted intercepts (estimated marginal means from the emmeans analysis) were tested based on Tukey’s HSD test. Scatter plots shown in the figures are based on raw (unadjusted) data, and all figure outputs were generated using Microsoft Excel.

## 3 Results

### 3.1 Morphological analysis of the lamina and petiole

**Figure 3:**
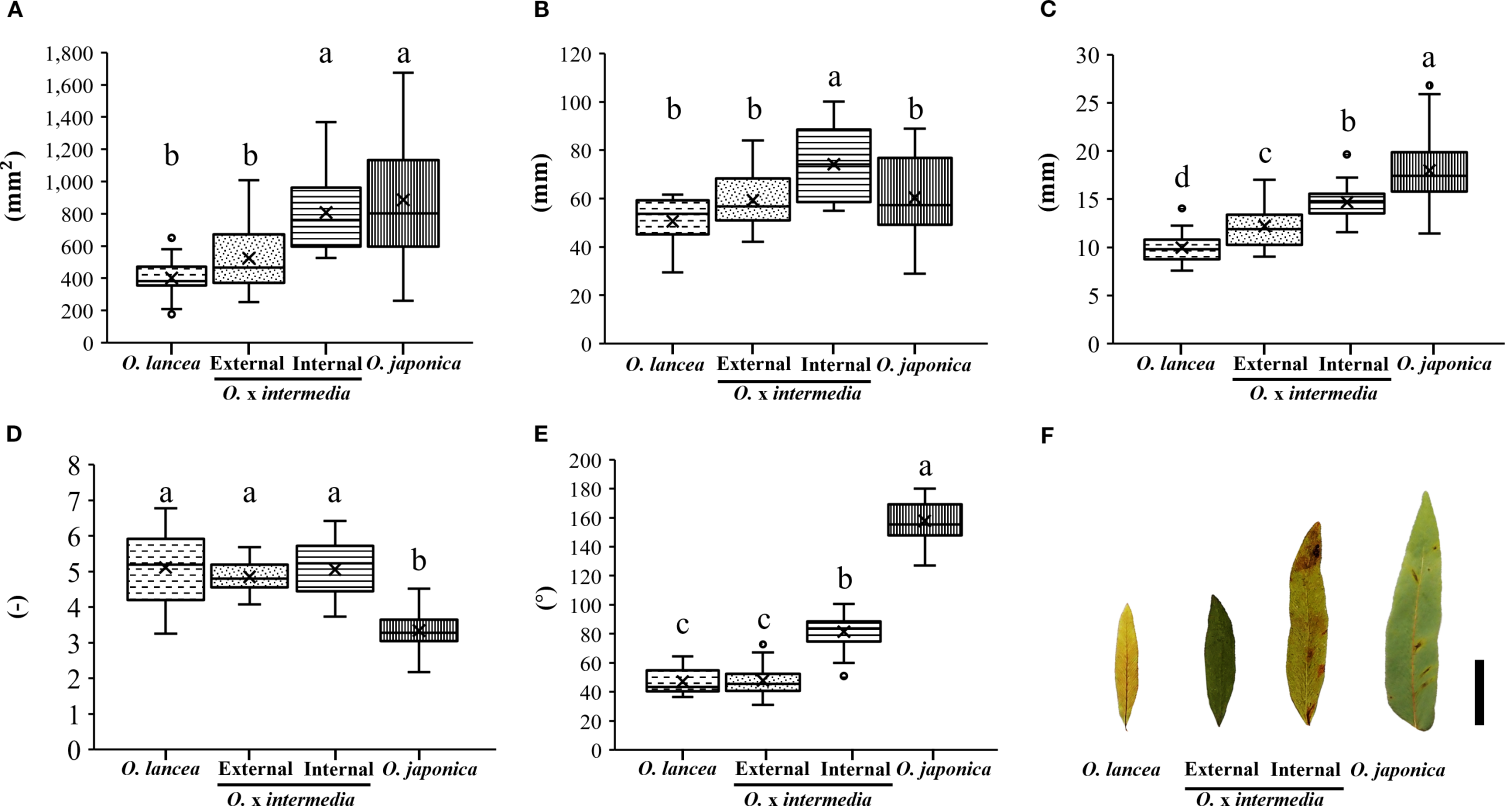
Comparative analyses of **(A)** pinnule area, **(B)** length, **(C)** width, **(D)** leaf index, and **(E)** angle at the base. Columns marked by different letters differ significantly according to the Tukey’s HSD test (*p* < 0.05). **(F)** Silhouette of a pinnule, scale bar = 2 cm.

**Figure 4:**
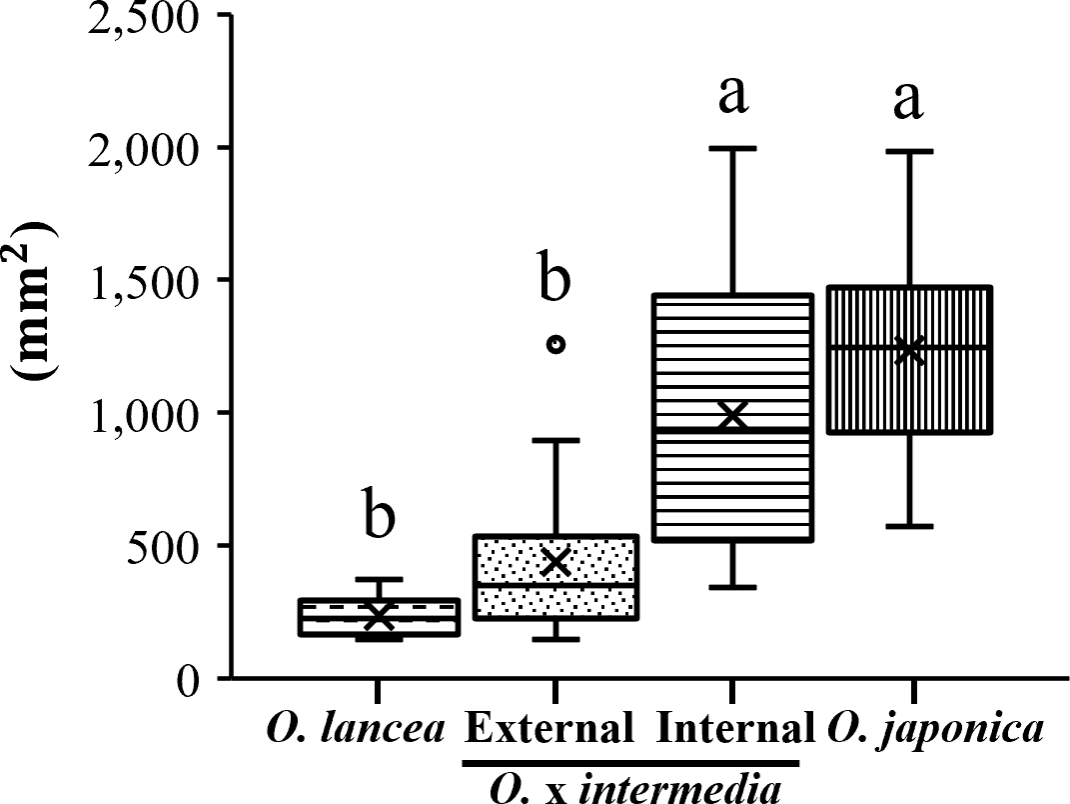
Comparative analysis of lamina area. Columns marked by different letters differ significantly according to the Tukey’s HSD test (*p* < 0.05).

Overall, *O. japonica* and the internal population exhibited larger pinnule and lamina dimensions compared with *O. lancea* and the external population (**Figure 3A**). Pinnule length was greatest in the internal population, whereas the other three groups showed similar values (**Figure 3B**). Pinnule width was widest in *O. japonica*, followed by the internal population, with the external population and *O. lancea* showing narrower pinnules (**Figure 3C**). Leaf index values were relatively high in *O. lancea* and both populations, but consistently lower in *O. japonica* (**Figure 3D**). The pinnule base angle was widest in *O. japonica* and moderately wide in the internal population, while the external population and *O. lancea* exhibited distinctly sharper bases (**Figure 3E**). The lamina area was larger in *O. japonica* and the internal population than in the external population and *O. lancea* (**Figures 3F**, **4**). These patterns indicate that *O. japonica* and the internal population share broader lamina traits, whereas *O. lancea* and the external population are characterized by narrower, more slender pinnules. These results indicate that *O. japonica* and the internal population share broad pinnule morphologies, which form larger laminae, whereas *O. lancea* and the external population are characterized by narrow and slender pinnules that form smaller laminae.

**Figure 5:**
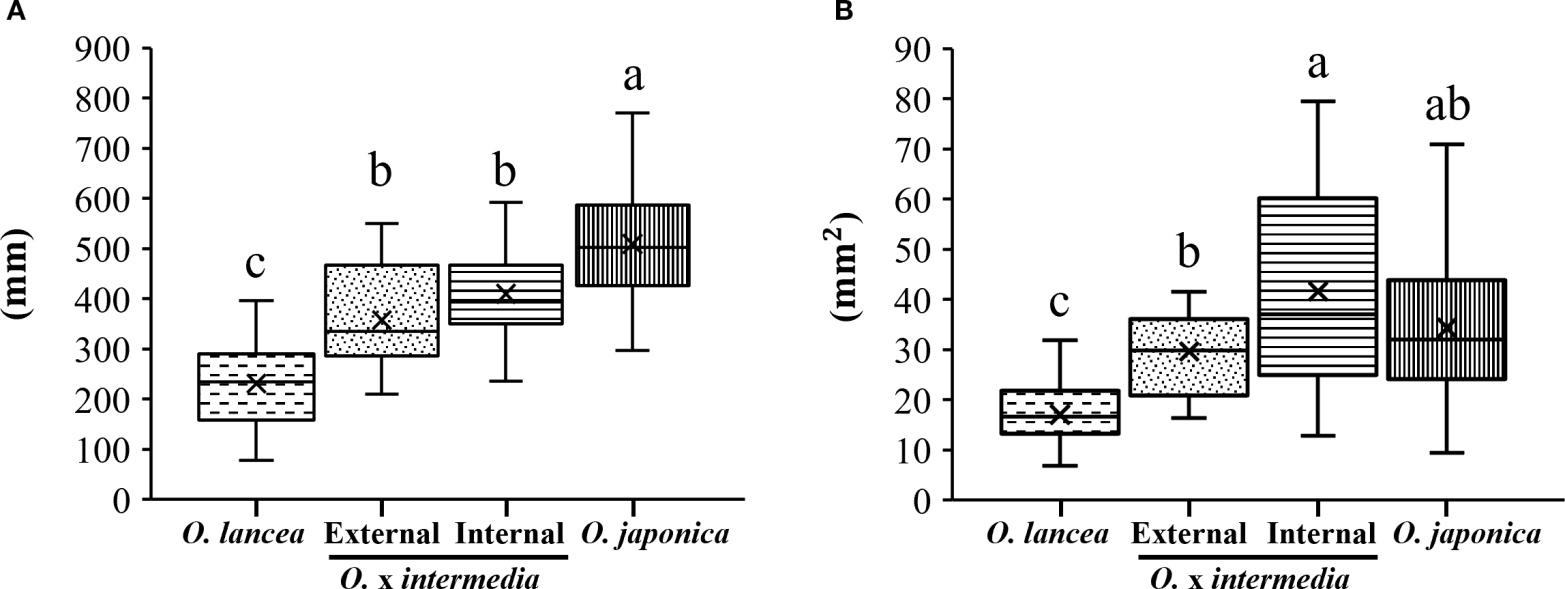
Comparative analyses of **(A)** petiole length and **(B)** cross-sectional area. Columns marked by different letters differ significantly according to the Tukey’s HSD test (*p* < 0.05).

Petiole length was greatest in *O. japonica* and shortest in *O. lancea*, while both populations showed intermediate values without differing from each other (**Figure 5A**). The petiole cross-sectional area was smaller in *O. lancea* than in both populations, whereas *O. japonica* exhibited an intermediate value similar to the hybrids (**Figure 5B**).

**Figure 6:**
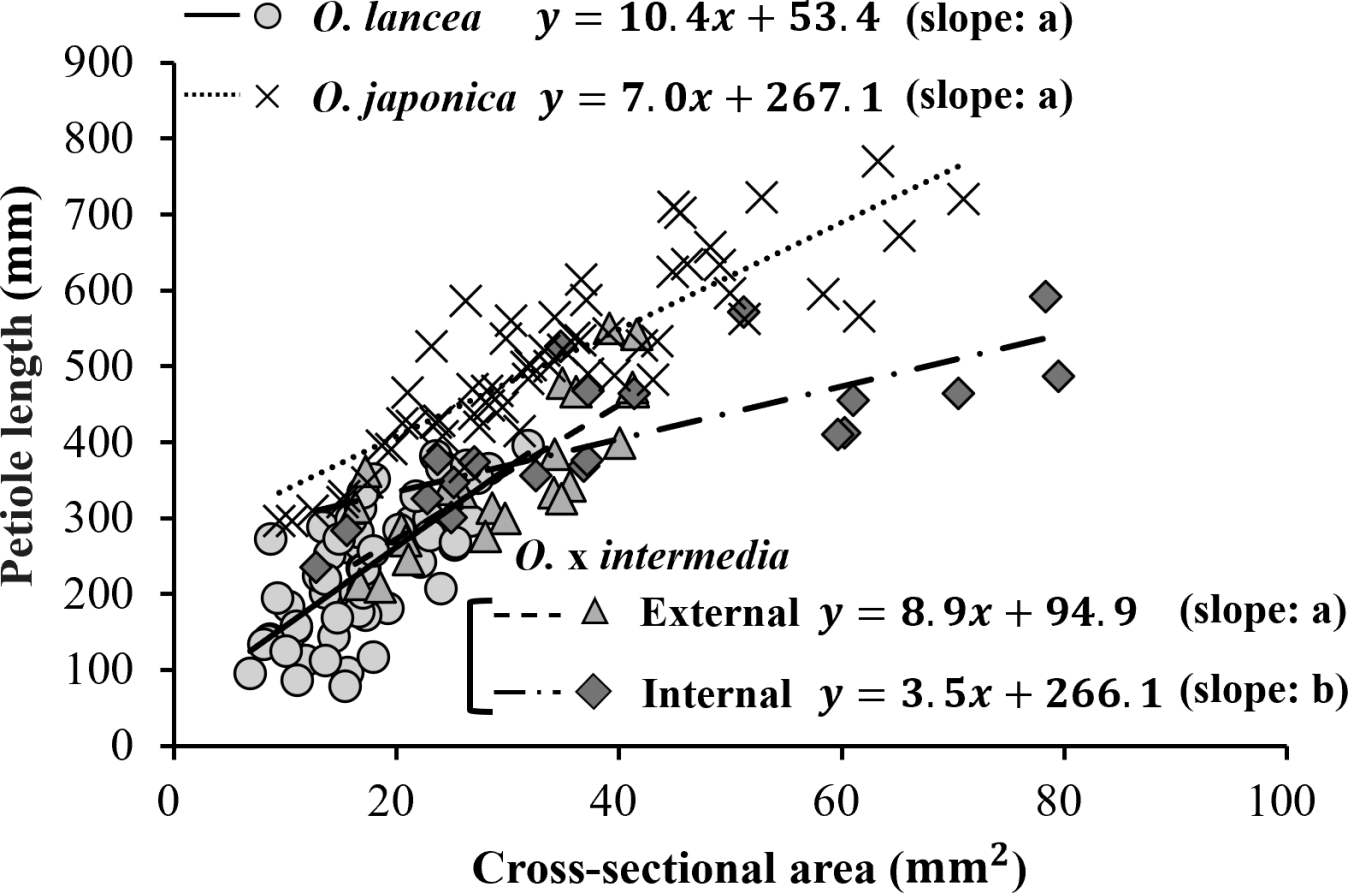
Relationship between petiole length and cross-sectional area. Significant differences in slopes among populations were tested using ANCOVA followed by Tukey’s HSD test (p< 0.05), and are indicated by different letters shown in parentheses in the figure. Statistical details and significance tests are described in the Results section.

A significant positive relationship was observed between petiole cross-sectional area and petiole length in all populations (*O. japonica*: r = 0.868, p< 0.001, n = 58; *O. lancea*: r = 0.713, p< 0.001, n = 58; internal population: r = 0.751, p< 0.001, n = 20; external population: r = 0.795, p< 0.001, n = 23; **Figure 6**). ANCOVA revealed a significant interaction between cross-sectional area and population (p< 0.001), indicating that slopes differed among populations. According to the emtrends analysis, the slope in the internal population (3.46 ± 0.67) was significantly lower than those in *O. japonica* (7.04 ± 0.56, p = 0.0004), *O. lancea* (10.42 ± 1.36, p = 0.0001), and the external population (8.87 ± 1.45, p = 0.0048). In contrast, no significant differences were detected among *O. japonica*, *O. lancea*, and the external population; the smallest non-significant result was p = 0.104. After adjusting for cross-sectional area using ANCOVA, the estimated marginal means of petiole length differed significantly among populations (emmeans analysis). *O. japonica* exhibited a greater adjusted petiole length (466 ± 8.5 mm) than the internal (364 ± 16.0 mm), external (345 ± 12.5 mm), and *O. lancea* populations (347 ± 17.1 mm), whereas no significant differences were detected among the latter three groups; the smallest non-significant result was p = 0.806 (Tukey’s HSD test).

**Figure 7:**
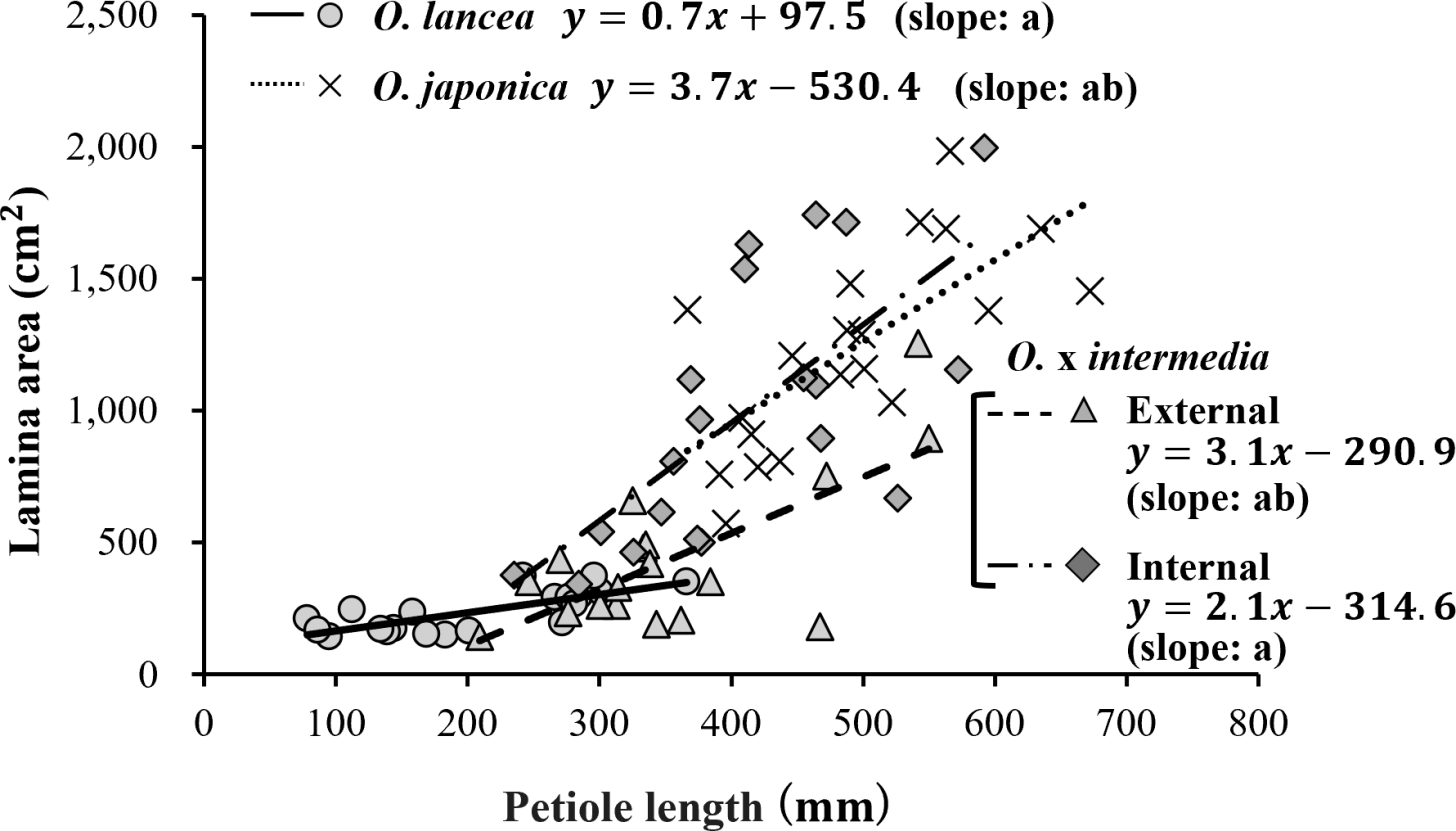
Relationship between petiole length and lamina area. Significant differences in slopes among populations were tested using ANCOVA followed by Tukey’s HSD test (p< 0.05), and are indicated by different letters shown in parentheses in the figure. Statistical details and significance tests are described in the Results section.

A significant positive relationship was observed between petiole length and lamina area in all populations (internal population: r = 0.685, p = 0.00085, n = 20; *O. japonica*: r = 0.704, p = 0.00054, n = 20; *O. lancea*: r = 0.749, p = 0.00015, n = 20; external population: r = 0.701, p = 0.00118, n = 18; **Figure 7**). ANCOVA revealed a significant interaction between petiole length and population (p = 0.0136), indicating that slopes differed among populations. According to the emtrends analysis, the slope in the internal population (3.71 ± 0.63) was significantly steeper than that in *O. lancea* (0.69 ± 0.70, p = 0.01), whereas no significant differences were detected among the other populations. After adjusting for petiole length using ANCOVA, the estimated marginal means of lamina area differed significantly among populations (emmeans analysis). *O. japonica* (842 ± 106 mm²) and the internal population (825 ± 64.5 mm²) exhibited greater adjusted lamina areas than the external (462 ± 61.6 mm²) and *O. lancea* populations (348 ± 127 mm²), whereas no significant differences were detected between *O. japonica* and the internal population (p = 0.99) or between the external and *O. lancea* populations (p = 0.84; Tukey’s HSD test).

### 3.2 Mechanical properties of the petiole

**Figure 8:**
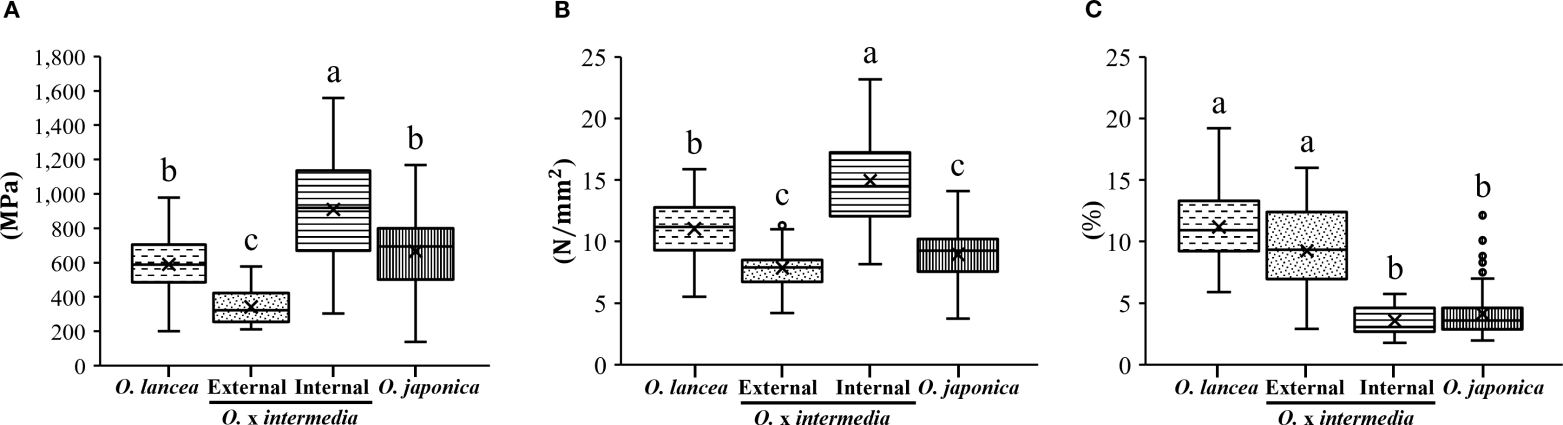
Comparative analyses of **(A)** bending modulus, **(B)** bending strength, and **(C)** breaking strain. Columns marked by different letters differ significantly according to the Tukey’s HSD test (*p* < 0.05).

**Figure 8** shows the results of the mechanical analysis of petioles. The bending modulus differed significantly among populations (p< 0.05), being the highest in the internal population and the lowest in the external population, while both parent species exhibited intermediate values with no significant difference between them (**Figure 8A**). The bending strength also varied significantly among populations (p< 0.05), with the internal population showing the highest value, *O. lancea* intermediate, and the other two populations the lowest (**Figure 8B**). The breaking strain was significantly higher in *O. lancea* and the external population than in the other populations (**Figure 8C**).

### 3.3 Cell wall volume per unit petiole volume and anatomical characteristics

**Figure 9:**
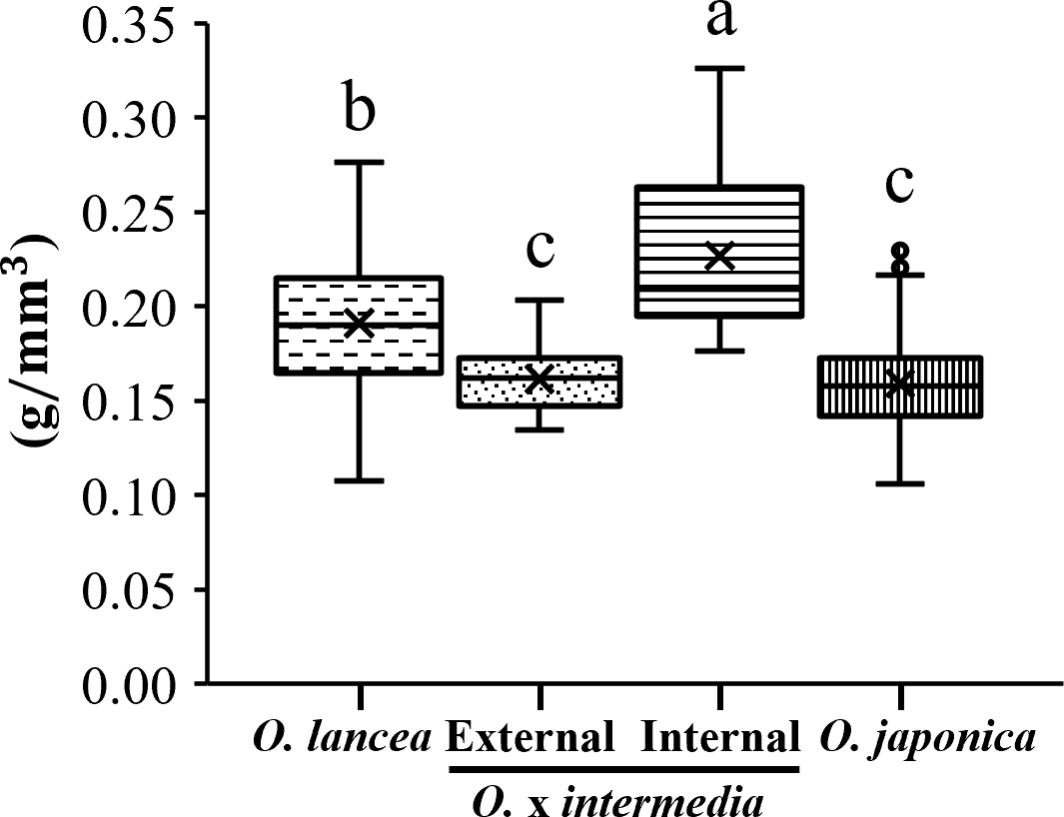
Comparative analyses of weight per unit volume in a petiole. Columns marked by different letters differ significantly according to the Tukey’s HSD test (*p* < 0.05).

The cell wall volume per unit petiole volume differed significantly among populations (p< 0.05), being the highest in the internal population, intermediate in *O. lancea*, and significantly lower in *O. japonica* and the external population, which did not differ from each other (**Figure 9**).

**Figure 10:**
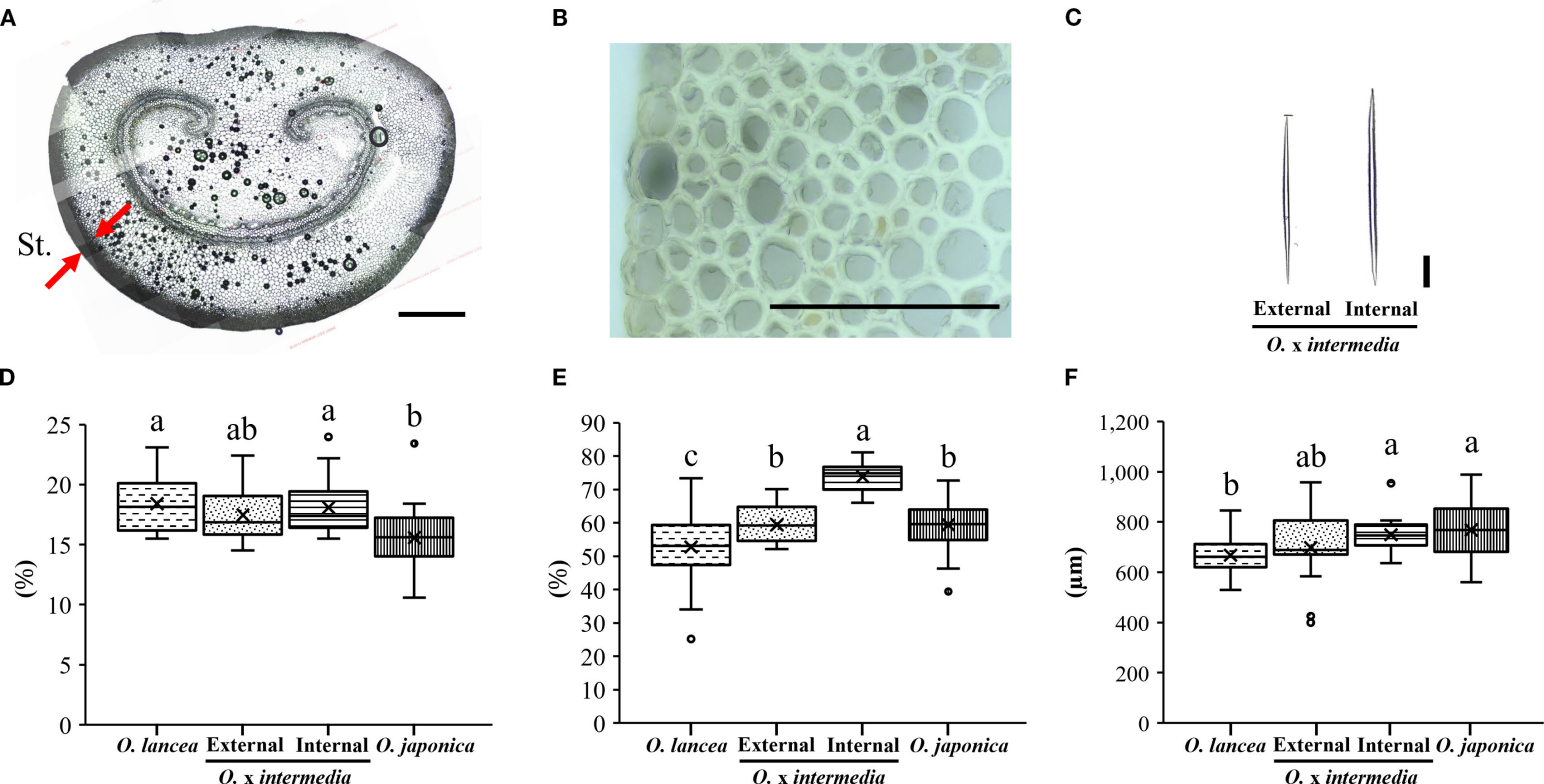
Anatomical analysis of the petiole. **(A)** Cross-section of a petiole, the red arrow indicates the sterome (St.), scale bar = 100 µm. **(B)** Structure of the cell wall of the sterome, and **(C)** isolated sclerenchyma cells observed from dissociated samples are shown for *Osmunda* x *intermedia*, scale bar = 100 µm. Comparisons were made for **(D)** sterome-to-petiole cross-sectional area ratio, **(E)** cell wall area fraction within the sterome, and **(F)** length of sclerenchyma cells. Columns marked by different letters differ significantly according to the Tukey’s HSD test (*p* < 0.05).

**Figure 10A** shows a cross-sectional image of the petiole of *O.* x *intermedia*. **Figures 10B, C** show the structures of the cell walls in the steromes observed in cross-sections and the sclerenchyma cells obtained from the macerated samples, respectively. The sterome-to-petiole cross-sectional area ratio was significantly higher in *O. lancea* and the internal population than in *O. japonica*, while the external population showed intermediate values without significant differences from any other population (**Figure 10D**). The cell wall area fraction within the sterome differed significantly among populations, being the highest in the internal population and the lowest in *O. lancea* (**Figure 10E**). The sclerenchyma cell length was significantly greater in *O. japonica* and the internal population than in *O. lancea*, whereas the external population exhibited intermediate values with no significant differences from the other populations (**Figure 10F**).

## 4 Discussion

### 4.1 Comparison of pinnule morphology on both banks

In general, the magnitude of mechanical forces experienced by plants exposed to strong winds and/or low water stresses is related to the plant size and shape, and the main morphological traits that allow them to avoid, reduce, and mitigate these forces are a small surface area exposed to fluids and a shape that lowers the forces experienced per unit area (Puijalon et al., 2005), the former is related to small growth forms and compact shapes (Speck, 2003; Rudnicki et al., 2004; Puijalon et al., 2005), whereas the latter is related to the shape of stems, leaves, and crowns (Telewski and Jaffe, 1986; Sand-Jensen, 2003; Puijalon et al., 2008). By contrast, the force required to break stems or petioles, which indicates the resistance of plants to mechanical forces, is expressed as the product of their cross-sectional area and material strength (Niklas, 1996), A large cross-sectional area of a plant organ or a high proportion of reinforcing tissue are plant traits that lead to high breakage resistance (Telewski and Jaffe, 1986; Ennos, 1997; Read and Stokes, 2006). Thus, various morphological variations are involved in mechanical forces, and hybrids exhibit a wide variety of traits which allow them to adapt to a wide range of environments (Rieseberg et al., 2000; Shiba et al., 2024c). Our results demonstrated that the size of the pinnule of *O.* x *intermedia* differed between the populations growing outside and inside the river curve, with the external populations being significantly smaller in pinnule size than the internal populations. To determine whether the pinnules were small or narrow based on these morphological results, Tsukaya (2002) proposed a leaf index value calculated as the ratio of leaf length to leaf width, which has been used in various studies to compare leaf silhouettes. Tsukaya (2002) found that the leaf indices of many riverside populations were significantly higher than those of neighboring populations, indicating that they had significantly thinner leaves (Yamada et al., 2011; Ohga et al., 2012a; Ueda et al., 2012; Yokoyama et al., 2012; Kumekawa et al., 2013; Matsui et al., 2013; Shiba and Fukuda, 2024). Our results revealed that the length and width of pinnules of *O.* x *intermedia* in the external population were significantly smaller than those in the internal population, and no significant differences were observed in the leaf index between them, indicating that the pinnules in the external population became smaller rather than narrower. Can plants adapt to water-flow stress by becoming smaller rather than narrower? Based on a comparison of leaf shapes between riverside and inland populations, Shiba et al. (2021) reported that the riverside populations of *Eurya japonica* Thunb. (Ternstroemiaceae) adapt to water-flow stress by developing relatively small leaves. The process of leaf miniaturization also included a reduction in width such that the population of *O.* x *intermedia* collected from the outer bank was able to adapt to strong water-flow stress by developing relatively small pinnules. In addition to the differences in pinnule size, a significant difference exists in the angle at the pinnule base between the two hybrid populations, indicating that the pinnule morphology of the external population was not simply smaller than that of the internal population but also included changes that resulted in a thinner base of the pinnule. This change in the angle at the leaf base is commonly observed in plant populations growing along rivers (Yamada et al., 2011; Ohga et al., 2012a; Ueda et al., 2012; Yokoyama et al., 2012; Kumekawa et al., 2013; Matsui et al., 2013; Shiba and Fukuda, 2024). Moreover, our results revealed that lamina size was smaller in the external population than in the internal population. Plants can prevent mechanical damage caused by external forces by creating structures that deflect resistance to large forces and reduce impact (Niklas, 1996; Read and Stokes, 2006). Whitehead (1962) reported that lamina size decreases with increasing mechanical stress. Our results also demonstrated a significant difference in lamina size between the two hybrid populations, suggesting that the decreased lamina area contributes to the reduced resistance to water flow, although it limits the amount of light available for photosynthesis. These results for the pinnule and lamina of *O.* x *intermedia* confirmed that the effects of water flow on plant traits are nonlinear, with a smaller effect on the internal population and a much larger effect on the external population, suggesting that the external population has experienced the history of stronger water-flow stress. Moreover, the contrasting gradient of increasing stress on the outer bend and decreasing stress on the inner bend of the river led to differential erosion and sediment deposition between both banks, the former being rocky while the latter was sandy at the survey site in our study. Therefore, these differences in soil conditions may also be involved in the aboveground morphology of *O.* x *intermedia.*

### 4.2 Morphological relationships between petioles and lamina under different water flow stresses

Plants are rooted in the soil and cannot move; therefore, they cannot respond to various abiotic stressors (van Loon, 2016). As plants are anchored at the base, mechanical stresses, such as water currents, in combination with the weights of leaves and reproductive organs, usually produce a bending moment in the stem and petiole, which is resisted by the bending strength that depends on their mechanical properties (Pinthus, 1974; Berry et al., 2004). For example, the shortening of stems and petioles can enhance the resistance of plant species against mechanical stresses (Cooper and Mendiola, 2004); however, our results demonstrated no significant difference in petiole length between the two hybrid populations. The internal population exhibited a significantly greater increase in lamina area relative to petiole length compared with *O. lancea* (**Figure 7**), but it was found to adopt a leaf morphology that reduces the bending moment caused by the weight of the large lamina by shortening petiole length relative to the basal cross-sectional area (**Figure 6**). This strategy appears to be specific to the internal population. By contrast, how can the external population adapt to strong water-flow stress without significant changes in petiole length? Our results revealed that the cross-sectional area of the base of the petiole in the external population of *O.* x *intermedia* was significantly smaller than that in the internal population. Moreover, the cross-sectional area of the petiole base and petiole length exhibited a correlation in both hybrid populations; however, the rate of increase was significantly different between the two populations. A comparative analysis of the results for *O. japonica* and *O. lancea* demonstrated that the growth rate of only the internal population differed significantly from that of the two parent species, and the external population was considerably similar to *O. lancea* in several characteristics, suggesting that even though no significant differences exist in petiole length between the two hybrid populations, the cross-sectional area of the petiole base in the external population exhibited morphological adaptations favoring its growth and survival along rivers. However, an intriguing question remains regarding how the petioles of the external populations of *O.* x *intermedia* support the lamina that are slightly larger than those of *O. lancea*, given the risk of petiole lodging—bending caused by external forces that results in a permanent deviation from vertical position, ultimately reducing reproduction success and fitness (Pinthus, 1974; Berry et al., 2004). Lodging is relatively more likely to occur in stems or petioles with small diameters and low strength (Islam et al., 2007). Furthermore, regarding the relationship between lamina area and petiole length, a significant difference in the slope was observed between *O. lancea* and the internal population of *O.* x *intermedia*. Although *O. lancea* tended to suppress the expansion of the lamina area with increasing petiole length, the external population exhibited a growth pattern wherein the lamina area increased relatively more actively with increasing petiole length. In this study, the relationship between petiole length and cross-sectional area and that between lamina area and petiole length exhibited different trends. Considering these differences in lamina and petiole morphology, an important question is how the mechanical properties of petioles, as supporting organs of the plant, are coordinated with these patterns.

### 4.3 Relationship between mechanical and anatomical characteristics of petioles

The stems and petioles of plants are exposed to a variety of forces, including their weight and additional external loads, such as wind, rain, snow, and animal movement; however, they have evolved to be strong, light, and can withstand damage without experiencing catastrophic failure (Speck and Burgert, 2011). The petiole of *Osmunda* performs various functions with conflicting requirements, such as orienting the lamina to the sun for photosynthesis, connecting the lamina with the vascular bundle, supporting the weight of the lamina, and elastic deflection of the leaf under water stress to prevent tearing (Shiba and Fukuda, 2024). In particular, achieving a mechanical compromise between high bending stiffness in the petiole to withstand the bending loads caused by the weight of the lamina and sufficient flexibility to avoid the damaging effects of water stress is important (Shiba and Fukuda, 2024). Regarding the mechanical analysis of petioles, the bending of the petiole increases with the bending moment. Because the petiole is elastic up to a certain limit, the plant quickly returns to an upright position once the bending force ceases (Pinthus, 1974). Beyond this limit, bending is irreversible and lodging occurs (Shah et al., 2017). Therefore, the maximum bending strength of the petiole is an important mechanical property of plant structure (Pinthus, 1974; Berry et al., 2004). The results of our mechanical analyses revealed that the petioles of the external population of *O. x intermedia* exhibited a significantly lower bending modulus and strength, but significantly higher breaking strain than those of the internal population, indicating that the petioles of the external population were more flexible and could deform to break more readily, although they could withstand a smaller maximum load than withstood by the petioles of the internal population. How did these mechanical differences arise in the petioles? Our anatomical analysis provides a hint towards understanding these differences. Plants have various types of cells and tissues which support the plant body structure. The epidermis of the stem is smooth and has a rather thick cuticle, but the outer cortical layer inside the epidermis is mainly hard and widespread, whereas the inner region is composed of thin-walled parenchyma tissue with flexible cellulose cell walls. The relative amounts of these two tissue types may importantly influence petiole flexibility under strong pressure; however, accurately measuring the properties of individual tissues and cell types remains challenging (Karam and Gibson, 1994). These tissues contain a lining in their walls which cannot be bent or broken, and the relative amounts of these two tissue types determine whether the plant will bend or stand under strong pressure (Moysset and Simón, 1991; Paiva and MaChado, 2003; Leroux, 2012). Our results for weight per unit volume in petioles added to *O. japonica* and *O. lancea* were similar to the bending modulus and strength, suggesting that the appreciation of the relative contribution of the cell walls to the mechanical properties of petioles in the genus *Osmunda* was also reflected in our experimental studies. Which petiolar tissues are mechanically important? Köhler and Spatz (2002) showed that the outer strengthening tissues have an elastic modulus and strength approximately four times higher than the core tissues, and Niklas and Paolillo (1997) also demonstrated that the mature epidermis is an important stiffening agent in turgid stems, indicating that the outer tissues are the principal structure supporting cells against tension and bending loads. Shiba and Fukuda (2024) suggested that the cell size and the relative amount of cell walls in the outer tissue of petioles play important roles in determining strength and flexibility. Therefore, we performed anatomical analyses of steromes in the external and internal populations of *O.* x *intermedia*. Although the proportion of the sterome in the petiole was not significantly different between the external and internal populations of *O*. x *intermedia*, the proportion of the cell wall in the sterome was significantly higher in the internal populations. Furthermore, no significant difference exists in stomatal cell height between the internal and external populations of *O.* x *intermedia*, suggesting that the external population possesses a thinner cell wall than the internal population. Therefore, the flexibility of the petioles of the external population was associated with a lower cell wall density in the sterome, which may cause elastic bending that bends the cells further because of the thinner cell walls. Thus, the resistance or avoidance of petioles and stems to external forces has been evaluated based on morphological measurements such as petiole length, cross-sectional area, and leaf blade size. However, our study showed that the combination of morphological, mechanical, and anatomical analyses could detect different petiole and stem characteristics, even between populations that appear to have similar external morphology. Hitherto, research has been conducted on the relationship between the lamina size in the Asteraceae plants *A. microcephalus* var. *ripensis* and *S. yambaruensis*, their river habitat, and the intensity of water-flow stress (Yamada et al., 2011; Sakaguchi et al., 2021); however, incorporating an analysis of supporting organs such as petioles and stems would allow for a detailed discussion of adaptation patterns that cannot be elucidated from lamina size alone.

## 5 Conclusions and future research

Hybridization and introgression, which can lead to hybridization and genome reticulation, are widely considered to be major evolutionary mechanisms that promote morphological change and adaptation in plants (Soltis and Soltis, 2009), and approximately 25% of plant species are known to hybridize with at least one other species (Mallet, 2005). Therefore, they play an important role in shaping plant biodiversity (Mallet, 2005; De Queiroz, 2007; Taylor and Larson, 2019). This study supported our hypothesis that the adaptive patterns of *O.* x *intermedia* differ on both sides of meandering rivers with different water flow stresses. Even more interestingly, our study revealed that the anatomical and mechanical traits of the external and internal populations of *O.* x *intermedia* did not necessarily exhibit values intermediate between those of the parents and that the combination of various characteristics contributed to lowering water-flow stress and enabled growth under different stresses. Our results also revealed that the intensity of river flow stress was reflected in the anatomical characteristics of petioles of *O.* x *intermedia*, which were not apparent from their external morphology, indicating that new anatomical features have been added to rheophytic plants, which have previously been characterized by morphological traits.

It is also intriguing to understand whether these morphological and anatomical traits of *O.* x *intermedia* are achieved through phenotypic plasticity or genetic variation. Cultivation experiments of each population will be effective in distinguishing them, leading to further research. Moreover, *O.* x *intermedia* has been reported throughout Japan (Shimura, 1964, 1972), possibly because it repeatedly hybridizes and backcrosses, creating opportunities for growth in diverse environments. In addition, Yatabe et al. (2011) reported that F_2_ and F_3_ offspring were formed in *O.* x *intermedia* under artificial conditions, suggesting that this species could be used as a model plant for the mechanical analysis of petioles. Further analyses using such populations are necessary to demonstrate the great diversity of *O.* x *intermedia*. However, soil erosion along riverbanks and riparian areas can lead to riverside areas instability and collapse (Chu-Agor et al., 2008, 2009; Daly et al., 2015) and increased sediment loads to rivers (Fox et al., 2016). Although plants along the river buffer trap nutrients and sediments from surface runoff and reduce the vulnerability of riverbanks to erosion (Sakio, 1997), it is unlikely that the current intensity of water-flow stress will continue in the future, and the *O.* x *intermedia* analyzed in this study may be replaced by populations with different traits to adapt to different water-flow stresses. Therefore, conducting long-term along-the-river surveys for *O.* x *intermedia* may help reveal the plant-induced changes in river conditions. Future investigations into the potential impacts of erosion and deposition processes on the riverbanks on both banks, as well as the possible influences of changes in soil properties and water saturation, will demonstrate that the complex bank-to-bank asymmetries common to many meandering rivers are suitable for analyzing quantitative changes in flow stress along the river.

## Data Availability

The datasets presented in this study can be found in online repositories. The names of the repository/repositories and accession number(s) can be found in the article/**Supplementary Material**.

## References

[B1] Allan J. D. Castillo M. M. (2007). Stream ecology: Structure and function of running waters, 2nd ed. (Dordrecht: Springer). doi: 10.1007/978-1-4020-5583-6

[B2] Allen J. R. L. (1965). A review of the origin and characteristics of recent alluvial sediments. Sedimentology. 5, 89–191. doi: 10.1111/j.1365-3091.1965.tb01561.x

[B3] Anest A. Charles-Dominique T. Maurin O. Millan M. Edelin C. Tomlinson K. W. (2021). Evolving the structure: climatic and developmental constraints on the evolution of plant architecture. A case study in *Euphorbia* . New Phytol. 231, 1278–1295. doi: 10.1111/nph.17296, PMID: 33629359

[B4] Berry P. M. Sterling M. Spink J. H. Baker C. J. Sylvester-Bradley R. Mooney S. J.. (2004). Understanding and reducing lodging in Cereals. Adv. Agron. 84, 217–271. doi: 10.1016/S0065-2113(04)84005-7

[B5] Blom C. W. P. M. Voesenek L. A. C. J. (1996). Flooding: the survival strategies of plants. Trends Ecol. Evol. 11, 290–295. doi: 10.1016/0169-5347(96)10034-3, PMID: 21237846

[B6] Carson M. A. Lapointe M. F. (1983). The inherent asymmetry of river meander planform. J. Geol. 91, 41–55. doi: 10.1086/628743

[B7] Chitale S. V. (1970). River channel patterns. J. Hydr. Div. (American Soc. Civil Engineers). 96, 201–221. doi: 10.1061/JYCEAJ.0002261

[B8] Chu-Agor M. L. Fox G. A. Wilson G. V. (2009). Empirical sediment transport function predicting seepage erosion undercutting for cohesive bank failure prediction. J. Hydrol. 377, 155–164. doi: 10.1016/j.jhydrol.2009.08.020

[B9] Chu-Agor M. L. Wilson G. V. Fox G. A. (2008). Numerical modeling of bank instability by seepage erosion undercutting of layered streambanks. J. Hydrol. Eng. 13, 1133–1145. doi: 10.1061/(ASCE)1084-0699(2008)13:12(1133

[B10] Cooper R. L. Mendiola T. (2004). Registration of 10 determinate semidwarf soybean germplasm lines. Crop Sci. 44, 699–700. doi: 10.2135/cropsci2004.6990

[B11] Cosmo N. L. Gogosz A. M. Botosso P. C. Kuniyoshi Y. S. Curcio G. R. (2024). Geopedological influence on the wood anatomy of *Gymnanthes klotzschiana* (Euphorbiaceae) in a subtropical riparian forest in southern Brazil. Plant Biosyst. 158, 511–522. doi: 10.1080/11263504.2024.2329460

[B12] Daly E. R. Miller R. B. Fox G. A. (2015). Modeling streambank erosion and failure along protected and unprotected composite streambanks. Adv. Water Resour. 81, 114–127. doi: 10.1016/j.advwatres.2015.01.004

[B13] Davies N. S. Shillito A. P. Slater B. J. Liu A. G. McMahon W. J. (2020). Evolutionary synchrony of Earth’s biosphere and sedimentary-stratigraphic record. Earth Sci. Rev. 201, 102979. doi: 10.1016/j.earscirev.2019.102979

[B14] de Langre E. (2008). Effects of wind on plants. Annu. Rev. Fluid Mech. 40, 141–168. doi: 10.1146/annurev.fluid.40.111406.102135

[B15] De Queiroz K. (2007). Species concepts and species delimitation. Syst. Biol. 56, 879–886. doi: 10.1080/10635150701701083, PMID: 18027281

[B16] Endo U. Shiba M. Fukuda T. (2025). The invasive alien species *Bidens pilosa* (Asteraceae) has successfully invaded and acclimated to coastal areas. Front. Conserv. Sci. 6. doi: 10.3389/fcosc.2025.1604666

[B17] Ennos A. R. (1997). Wind as an ecological factor. Trends Ecol. Evol. 12, 108–111. doi: 10.1016/S0169-5347(96)10066-5, PMID: 21237994

[B18] Erskine W. Chalmers A. Keene A. Cheetham M. Bush R. (2009). Role of a rheophyte in bench development on a sand-bed river in southeast Australia. Earth Surf. Process. Landforms. 34, 941–953. doi: 10.1002/esp.1778

[B19] Fox G. A. Purvis R. A. Penn C. J. (2016). Streambanks: A net source of sediment and phosphorus to streams and rivers. J. Environ. Manage. 181, 602–614. doi: 10.1016/j.jenvman.2016.06.071, PMID: 27429360

[B20] Gardiner B. Berry P. Moulia B. (2016). Review: wind impacts on plant growth, mechanics and damage. Plant Sci. 245, 94–118. doi: 10.1016/j.plantsci.2016.01.006, PMID: 26940495

[B21] Gibling M. R. Davies N. S. (2012). Palaeozoic landscapes shaped by plant evolution. Nat. Geosci. 5, 99–105. doi: 10.1038/ngeo1376

[B22] Givnish T. J. (2015). Adaptive radiation versus “radiation” and “explosive diversification”: why conceptual distinctions are fundamental to understanding evolution. New Phytol. 207, 297–303. doi: 10.1111/nph.13482, PMID: 26032979

[B23] Grant P. R. (1981). Speciation and the adaptive radiation of Darwin’s finches: the complex diversity of Darwin’s finches may provide a key to the mystery of how intraspecific variation is transformed into interspecific variation. Am. Sci. 69, 653–663. Available online at: https://www.jstor.org/stable/27850717.

[B24] Hayakawa H. Tunala M. Y. Minamiya Y. Ito K. Gale S. Yokoyama J.. (2012). Comparative Study of leaf morphology in *Aster hispidus* Thunb. var. *leptocladus* (Makino) Okuyama (Asteraceae). Am. J. Plant Sci. 3, 110–113. doi: 10.4236/ajps.2012.31011

[B25] Imaichi R. Kato M. (1992). Comparative leaf development of *Osmunda lancea* and O. japonica (Osmundaceae): heterochronic origin of rheophytic stenophylly. Bot. Mag. Tokyo. 105, 199–213. doi: 10.1007/BF02489415

[B26] Imaichi R. Kato M. (1993). Comparative leaf morphology of young sporophytes of rheophytic *Osmunda lancea* and dryland O. japonica. J. Plant Res. 106, 37–45. doi: 10.1007/BF02344371

[B27] Ishii C. Shiba M. Kumekawa Y. Fukuda T. (2022). Seed germination and seedling emergence of *Canavalia lineata* (Thunb.) DC. (Fabaceae). Int. J. Biol. 14, 8–18. doi: 10.5539/ijb.v14n1p8

[B28] Islam M. S. Peng S. Visperas R. M. Ereful N. Bhuiya M. S. U. Julfiquar A. W. (2007). Lodging-related morphological traits of hybrid rice in a tropical irrigated ecosystem. Field Crops Res. 101, 240–248. doi: 10.1016/j.fcr.2006.12.002

[B29] Iwatsuki K. (1995). “Osmundaceae,” in Flora of Japan, vol. 1 . Ed. Iwatsuki K.. (Kodansha, Tokyo), 31–33.

[B30] Karam G. N. Gibson L. J. (1994). Biomimicking of animal quills and plant stems: natural cylindrical shells with foam cores. Mater. Sci. Eng. C. 2, 113–132. doi: 10.1016/0928-4931(94)90039-6

[B31] Kato M. (2007). Distribution of osmundaceae. Bull. Natl. Mus. Nat. Sci. Ser. B Bot. 33, 81–90.

[B32] Kijima M. (1987). Laboratory Manual of Botany (Tokyo: Hirokawa Publishing Co).

[B33] Kinoshita R. Miwa H. (1974). River channel formation which prevents downstream translation of transverse bars. Shinsabo. 94, 12–17.

[B34] Köhler L. Spatz H. C. (2002). Micromechanics of plant tissues beyond the linear-elastic range. Planta. 215, 33–40. doi: 10.1007/s00425-001-0718-9, PMID: 12012239

[B35] Kumekawa Y. Miyata H. Ohga K. Hayakawa H. Yokoyama J. Ito K.. (2013). Comparative analyses of stomatal size and density among ecotypes of *Aster hispidus* (Asteraceae). Am. J. Plant Sci. 4, 524–527. doi: 10.4236/ajps.2013.43067

[B36] Leopold L. B. Wolman M. G. (1960). River meanders. Geol. Soc America Bull. 71, 769–794. doi: 10.1130/0016-7606(1960)71[769:RM]2.0.CO;2

[B37] Leroux O. (2012). Collenchyma: a versatile mechanical tissue with dynamic cell walls. Ann. Bot. 110, 1083–1098. doi: 10.1093/aob/mcs186, PMID: 22933416 PMC3478049

[B38] Louf J. F. Nelson L. Kang H. Song P. N. Zehnbauer T. Jung S. (2018). How wind drives the correlation between leaf shape and mechanical properties. Sci. Rep. 8, 16314. doi: 10.1038/s41598-018-34588-0, PMID: 30397247 PMC6218545

[B39] Mallet J. (2005). Hybridization as an invasion of the genome. Trends Ecol. Evol. 20, 229–237. doi: 10.1016/j.tree.2005.02.010, PMID: 16701374

[B40] Marui Y. Takizawa E. Shiba M. Yoshizaki S. Fukuda T. (2023). Seed germination and seedling emergence of *Lysimachia mauritiana* Lam. (Primulaceae). Int. J. Biol. 15, 13–23. doi: 10.5539/ijb.v15n1p13

[B41] Matsui R. Takei S. Ohga K. Hayakawa H. Yoshida M. Yokoyama J.. (2013). Morphological and anatomical variations in rheophytic ecotype of violet, *Viola mandshurica* var. *ikedaeana* (Violaceae). Am. J. Plant Sci. 4, 859–865. doi: 10.4236/ajps.2013.44106

[B42] Meguro S. Miyawaki A. (1994). A study of the relationship between mechanical characteristics and the coastal vegetation among several broad-leaf trees in Miura Peninsula in Japan. Vegetatio. 112, 101–111. doi: 10.1007/BF00044685

[B43] Mitchell S. J. (2013). Wind as a natural disturbance agent in forests: a synthesis. Forestry. 86, 147–157. doi: 10.1093/forestry/cps058

[B44] Moysset L. Simón E. (1991). Secondary pulvinus of *Robinia pseudoacacia* (Leguminosae)- structural and ultrastructural features. Am. J. Bot. 78, 1467–1486. doi: 10.1002/j.1537-2197.1991.tb11426.x

[B45] Nanson G. C. Hickin E. J. (1983). Channel migration and incision on the Beatton River. J. Hydraul. Eng. 109, 327–337. doi: 10.1061/(ASCE)0733-9429(1983)109:3(327

[B46] Niklas K. J. (1996). Differences between *Acer saccharum* leaves from open and wind-protected sites. Ann. Bot. 78, 61–66. doi: 10.1006/anbo.1996.0096

[B47] Niklas K. J. Paolillo D. J. (1997). The role of the epidermis as a stiffening agent in *Tulipa* (Liliaceae) stems. Am. J. Bot. 84, 735. doi: 10.2307/2445809, PMID: 21708626

[B48] Ohga K. Muroi M. Hayakawa H. Yokoyama J. Ito K. Tebayashi S. I.. (2012a). Comparative morphology and anatomy of non-rheophytic and rheophytic types of *Adenophora triphylla* var. *japonica* (Campanulaceae). Am. J. Plant Sci. 3, 805–809. doi: 10.4236/ajps.2012.36097

[B49] Ohga K. Muroi M. Hayakawa H. Yokoyama J. Ito K. Tebayashi S.. (2012b). Morphological and anatomical analyses of the serpentine ecotype of Adenophora triphylla var. *japonica* (Campanulaceae). J. Plant Stud. 1, 180–187. doi: 10.5539/jps.v1n2p180

[B50] Paiva E. A. S. MaChado S. R. (2003). Collenchyma in *Panicum maximum* (Poaceae): localisation and possible role. Aust. J. Bot. 51, 69–73. doi: 10.1071/BT02046

[B51] Pinthus M. J. (1974). Lodging in wheat, barley, and oats: the phenomenon, its causes, and preventive measures. Adv. Agron. 25, 209–263. doi: 10.1016/S0065-2113(08)60782-8

[B52] Puijalon S. Bornette G. Sagnes P. (2005). Adaptations to increasing hydraulic stress: morphology, hydrodynamics and fitness of two higher aquatic plant species. J. Exp. Bot. 56, 777–786. doi: 10.1093/jxb/eri063, PMID: 15642713

[B53] Puijalon S. Léna J. P. Rivière N. Champagne J. Y. Rostan J. C. Bornette G. (2008). Phenotypic plasticity in response to mechanical stress: hydrodynamic performance and fitness of four aquatic plant species. New Phytol. 177, 907–917. doi: 10.1111/j.1469-8137.2007.02314.x, PMID: 18275493

[B54] Read J. Stokes A. (2006). Plant biomechanics in an ecological context. Am. J. Bot. 93, 1546–1565. doi: 10.3732/ajb.93.10.1546, PMID: 21642101

[B55] Rieseberg L. H. Baird S. J. Gardner K. A. (2000). Hybridization, introgression, and linkage evolution. Plant Mol. Biol. 42, 205–224. doi: 10.1023/A:1006340407546 10688138

[B56] Rowe N. Speck T. (2005). Plant growth forms: an ecological and evolutionary perspective. New Phytol. 166, 61–72. doi: 10.1111/j.1469-8137.2004.01309.x, PMID: 15760351

[B57] Rudnicki M. Mitchell S. J. Novak M. D. (2004). Wind tunnel measurements of crown streamlining and drag relationships for three conifer species. Can. J. For. Res. 34, 666–676. doi: 10.1139/x03-233

[B58] Sakaguchi S. Abe A. Nagasawa K. Takahashi D. Setoguchi H. Maki M.. (2021). Functional traits divergence in parallelly evolved rheophytic populations of *Solidago virgaurea* L. complex (Asteraceae) in Japan. Acta Phytotaxon. Geobot. 72, 93–111. doi: 10.18942/apg.202012

[B59] Sakio H. (1997). Effects of natural disturbance on the regeneration of riparian forests in a Chichibu Mountains, central Japan. Plant Ecol. 132, 181–195. doi: 10.1023/A:1009775923208

[B60] Salo J. Kalliola R. Häkkinen I. Mäkinen Y. Niemelä P. Puhakka M.. (1986). River dynamics and the diversity of Amazon lowland forest. Nature. 322, 254–258. doi: 10.1038/322254a0

[B61] Sand-Jensen K. (2003). Drag and reconfiguration of freshwater macrophytes. Freshw. Biol. 48, 271–283. doi: 10.1046/j.1365-2427.2003.00998.x

[B62] Santiago L. S. Wright S. J. (2007). Leaf functional traits of tropical forest plants in relation to growth form. Funct. Ecol. 21, 19–27. doi: 10.1111/j.1365-2435.2006.01218.x

[B63] Seminara G. (1998). Stability and morphodynamics. Meccanica. 33, 59–99. doi: 10.1023/A:1004225516566

[B64] Seminara G. (2006). Meanders. J. Fluid Mech. 554, 271–297. doi: 10.1017/S0022112006008925

[B65] Shah D. U. Reynolds T. P. S. Ramage M. H. (2017). The strength of plants: theory and experimental methods to measure the mechanical properties of stems. J. Exp. Bot. 68, 4497–4516. doi: 10.1093/jxb/erx245, PMID: 28981787

[B66] Shiba M. Arihara S. Harada S. Fukuda T. (2024a). Impact on the scape of *Farfugium japonicum* var. *japonicum* (Asteraceae) under strong wind conditions based on morphological and mechanical analyses. Front. Plant Sci. 15. doi: 10.3389/fpls.2024.1407127, PMID: 39166247 PMC11333370

[B67] Shiba M. Fukuda T. (2024). Rheophytic *Osmunda lancea* (Osmundaceae) exhibits large flexibility in the petiole. Sci. Rep. 14, 2866. doi: 10.1038/s41598-024-53406-4, PMID: 38311628 PMC10838915

[B68] Shiba M. Fukuda T. (2025). Mechanical flexibility of fertile frond stipes in the rheophytic fern *Osmunda lancea* . Sci. Rep. 15, 29664. doi: 10.1038/s41598-025-15715-0, PMID: 40804437 PMC12350940

[B69] Shiba M. Kobayashi N. Harada S. Fukuda T. (2024b). Decrease in wind stress leads to an increase in the above ground morphology and number of seeds of an invasive alien species, *Bidens pilosa* (Asteraceae). Front. Plant Sci. 15. doi: 10.3389/fpls.2024.1445437, PMID: 39582631 PMC11581870

[B70] Shiba M. Mizuno T. Fukuda T. (2023). Effect of strong wind on laminas and petioles of *Farfugium japonicum* (L.) Kitam. var. *japonicum* (Asteraceae). Front. Plant Sci. 14. doi: 10.3389/fpls.2023.1182266, PMID: 37457339 PMC10345509

[B71] Shiba M. Sato R. Fukuda T. (2024c). A comparison of mechanical characteristics among *Setaria viridis* var. minor, *Setaria italica*, and *Setaria* x *Pycnocoma* species of the family Poaceae. Plant Species Biol. 39, 51–58. doi: 10.1111/1442-1984.12435

[B72] Shiba M. Tate T. Fukuda T. (2021). Rheophytic adaptation of *Eurya japonica* Thunb. (Ternstroemiaceae). Int. J. Biol. 13, 65–73. doi: 10.5539/ijb.v13n2p65

[B73] Shiba M. Tate T. Fukuda T. (2022a). Adaptative leaf morphology of *Eurya japonica* Thunb. (Ternstroemiaceae) in serpentine areas. J. Plant Stud. 11, 10–18. doi: 10.5539/jps.v11n1p10

[B74] Shiba M. Tate T. Fukuda T. (2022b). Leaf adaptation of eurya japonica thunb. (Pentaphylacaceae) in coastal area. J. Plant Stud. 11, 31–41. doi: 10.5539/jps.v11n1p31

[B75] Shiba M. Tate T. Fukuda T. (2022c). Serpentine adaptation of *Ligustrum japonicum* Thunb. (Oleaceae) based on morphological and anatomical approaches. Int. J. Biol. 14, 10–18. doi: 10.5539/ijb.v14n2p10

[B76] Shimura Y. (1964). Observations on the fertile fronds of *Osmunda lancea* var. *latipinnula* . J. Jpn. Bot. 39, 242–246.

[B77] Shimura Y. (1972). Study of reproduction of *Osmunda* x *intermedia* Sugimoto. Acta Phytotaxon. Geobot. 20, 38–42.

[B78] Soltis P. S. Soltis D. E. (2009). The role of hybridization in plant speciation. Annu. Rev. Plant Biol. 60, 561–588. doi: 10.1146/annurev.arplant.043008.092039, PMID: 19575590

[B79] Speck O. (2003). Field measurements of wind speed and reconfiguration in *Arundo donax* (Poaceae) with estimates of drag forces. Am. J. Bot. 90, 1253–1256. doi: 10.3732/ajb.90.8.1253, PMID: 21659225

[B80] Speck T. Burgert I. (2011). Plant stems: functional design and mechanics. Annu. Rev. Mater. Res. 41, 169–193. doi: 10.1146/annurev-matsci-062910-100425

[B81] Stella J. C. Hayden M. K. Battles J. J. Piégay H. Dufour S. Fremier A. K. (2011). The role of abandoned channels as refugia for sustaining pioneer riparian forest ecosystems. Ecosystems 14, 776–790. doi: 10.1007/s10021-011-9446-6

[B82] Sunami T. Ohga K. Muroi M. Hayakawa H. Yokoyama J. Ito K.. (2013). Comparative analyses of hairless-leaf and hairy-leaf type individuals in *Aster hispidus* var. *insularis* (Asteraceae). J. Plant Stud. 2, 1–6. doi: 10.5539/jps.v2n1p1

[B83] Swanson D. C. (1993). The importance of fluvial processes and related reservoir deposits. J. Petrol. Technol. 45, 368–377. doi: 10.2118/23722-PA

[B84] Takizawa E. Shiba M. Yoshizaki S. Fukuda T. (2023). Stomatal study of introduced species, *Ligustrum lucidum* Aiton (Oleaceae), in Coastal Areas of Japan. J. Plant Stud. 12, 24–36. doi: 10.5539/jps.v12n1p24

[B85] Takizawa E. Tate T. Shiba M. Ishii C. Yoshizaki S. Fukuda T. (2022). Coastal adaptation of *Ligustrum japonicum* Thunb. (Oleaceae). J. Jpn. Soc Coast. Forest. 21, 1–8. doi: 10.60398/kaiganrin.21.1_1

[B86] Taylor S. A. Larson E. L. (2019). Insights from genomes into the evolutionary importance and prevalence of hybridization in nature. Nat. Ecol. Evol. 3, 170–177. doi: 10.1038/s41559-018-0777-y, PMID: 30697003

[B87] Telewski F. W. Jaffe M. J. (1986). Thigmomorphogenesis: field and laboratory studies of *Abies fraseri* in response to wind or mechanical perturbation. Physiol. Plant 66, 211–218. doi: 10.1111/j.1399-3054.1986.tb02411.x, PMID: 11538654

[B88] Thorne S. D. Furbish D. J. (1995). Influences of coarse bank roughness on flow within a sharply curved river bend. Geomorphology. 12, 241–257. doi: 10.1016/0169-555X(95)00007-R

[B89] Tsukaya H. (2002). The leaf index: heteroblasty, natural variation, and the genetic control of polar processes of leaf expansion. Plant Cell Physiol. 43, 372–378. doi: 10.1093/pcp/pcf051, PMID: 11978864

[B90] Tsutsumi C. Hirayama Y. Yamamoto K. Kato H. Murakami N. Tsukaya H.. (2013). Hybrid of *osmunda japonica* and O. Lancea on mt. Tenjo, Kozu Island, Izu Islands, Japan. Bull. Natl. Mus. Nat. Sci. Ser. B Bot. 41, 99–105.

[B91] Tunala H. H. Minamiya Y. Gale S. W. Yokoyama J. Arakawa R. Fukuda T. (2012). Foliar adaptations in *Aster hispidus* var. *insularis* (Asteraceae). J. Plant Stud. 1, 19–25. doi: 10.5539/jps.v1n2p19

[B92] Ueda R. Minamiya Y. Hirata A. Hayakawa H. Muramatsu Y. Saito M.. (2012). Morphological and anatomical analyses of rheophytic *Rhododendron ripense* Makino (Ericaceae). Plant Species Biol. 27, 233–240. doi: 10.1111/j.1442-1984.2011.00345.x

[B93] van Eck W. H. J. M. van de Steeg H. M. Blom C. W. P. M. de Kroon H. (2004). Is tolerance to summer flooding correlated with distribution patterns in river floodplains? A comparative study of 20 terrestrial grassland species. Oikos. 107, 393–405. doi: 10.1111/j.0030-1299.2004.13083.x

[B94] van Loon L. C. (2016). The intelligent behavior of plants. Trends Plant Sci. 21, 286–294. doi: 10.1016/j.tplants.2015.11.009, PMID: 26690331

[B95] van Steenis C. G. G. J. (1981). Rheophyte of the World Vol. 68 (Alpen Aan Den Rijn: Sijthoff and Noordhoff), 143–144. doi: 10.1002/iroh.19830680115

[B96] Vervuren P. J. A. Blom C. W. P. M. de Kroon H. (2003). Extreme flooding events on the Rhine and the survival and distribution of riparian plant species. J. Ecol. 91, 135–146. doi: 10.1046/j.1365-2745.2003.00749.x

[B97] Whitehead F. H. (1962). Experimental studies of the effect of wind on plant growth and anatomy II. *Helianthus annuus* . New Phytol. 61, 59–62. doi: 10.1111/j.1469-8137.1962.tb06274.x

[B98] Whiting P. J. Dietrich W. E. (1993a). Experimental studies of bed topography and flow patterns in large-amplitude meanders: 1. Observations. Water Resour. Res. 29, 3605–3614. doi: 10.1029/93WR01755

[B99] Whiting P. J. Dietrich W. E. (1993b). Experimental studies of bed topography and flow patterns in large-amplitude meanders: 2. Mechanisms. Water Resour. Res. 29, 3615–3622. doi: 10.1029/93WR01756

[B100] Yamada Y. Hayakawa H. Minamiya Y. Ito K. Shibayama Z. Arakawa R.. (2011). Comparative morphology and anatomy of rheophytic *Aster microcephalus* (Miq.) Franch. et Sav. Var. *Ripensis* Makino (Asteraceae). J. Phytogeogr. Taxon. 59, 35–42. doi: 10.24517/00053453

[B101] Yatabe Y. Nishida H. Murakami N. (1999). Phylogeny of Osmundaceae inferred from *rbc*L nucleotide sequences and comparison to the fossil evidences. J. Plant Res. 112, 397–404. doi: 10.1007/PL00013894

[B102] Yatabe Y. Yamamoto K. Tsutsumi C. Shinohara W. Murakami N. Kato M. (2011). Fertility and precocity of *Osmunda x intermedia* offspring in culture. J. Plant Res. 124, 265–268. doi: 10.1007/s10265-010-0374-x, PMID: 20839027

[B103] Yokoyama N. Hayakawa H. Matsuyama K. Muroi M. Ohga K. Ito K.. (2012). Morphological and molecular analyses of rheophytic *Rhododendron ripense* and its allied dryland species *R. macrosepalum* (Ericaceae). Environ. Control Biol. 50, 305–312. doi: 10.2525/ecb.50.305

[B104] Zimmerman C. Kennedy J. F. (1978). Transverse bed slopes in curved alluvial streams. J. Hydr. Div. (American Soc. Civil Engineers). 104, 33–48. doi: 10.1061/JYCEAJ.0004922

